# Fabrication, characterization and simulation of Ω-gate twin poly-Si FinFET nonvolatile memory

**DOI:** 10.1186/1556-276X-8-331

**Published:** 2013-07-22

**Authors:** Mu-Shih Yeh, Yung-Chun Wu, Min-Feng Hung, Kuan-Cheng Liu, Yi-Ruei Jhan, Lun-Chun Chen, Chun-Yen Chang

**Affiliations:** 1 Department of Engineering and System Science, National Tsing Hua University, 101, Section 2 Kuang Fu Road, Hsinchu, 30013, Taiwan; 2 Department of Electronics Engineering and Institute of Electronics, National Chiao Tung University, 1001, Ta Hsueh Road, Hsinchu, 30013, Taiwan

**Keywords:** Twin poly-Si, FinFET, TFT, Nonvolatile memory, Ω-gate, Nanowires, Three-dimensional, Flash memory

## Abstract

This study proposed the twin poly-Si fin field-effect transistor (FinFET) nonvolatile memory with a structure that is composed of Ω-gate nanowires (NWs). Experimental results show that the NW device has superior memory characteristics because its Ω-gate structure provides a large memory window and high program/erase efficiency. With respect to endurance and retention, the memory window can be maintained at 3.5 V after 10^4^ program and erase cycles, and after 10 years, the charge is 47.7% of its initial value. This investigation explores its feasibility in the future active matrix liquid crystal display system-on-panel and three-dimensional stacked flash memory applications.

## Background

Electrically erasable programmable read-only memory (EEPROM), which is a kind of nonvolatile memory (NVM) [1,2], has been widely used in portable products owing to its high density and low cost [3]. Embedded EEPROM that is based on poly-Si thin film transistor (TFT) has attracted much attention because it can meet the low-temperature process requirement in thin film transistor liquid crystal display applications [4,5]. However, since the process and physical limitations of the device limit the scaling of the flash NVM that is based on a single-crystalline Si substrate, according to Moore’s law, the three-dimensional (3D) multi-layer stack memory provides a high-density flash memory solution. The poly-Si-based NVM also has great potential for realizing 3D high-density multi-layer stack memory [6-8]. A planar EEPROM that uses twin poly-Si TFTs has also been developed for the above aforementioned applications [4,9]. The advantages of this twin TFT structure include processing identical to that of a conventional TFT, which is easily embedded on Si wafer, glass, and flexible substrates. Additionally, the low program/erase (P/E) operating voltage of this planar NVM can be easily obtained by increasing the artificial gate coupling ratio (*α*_G_).

Recently, several investigations have demonstrated that gate control can be substantially enhanced by introducing a multi-gate with a nanowire (NW) structure [10-12]. In our previous works [13,14], NWs were introduced into twin poly-Si TFT NVM to increase P/E speed. However, reducing the P/E voltage while ensuring the reliability of this device remains a challenge.

Therefore, in this work, to reduce the P/E voltage, we try to use p-channel devices with band-to-band tunneling-induced hot electron (BBHE) operation compared with Fowler-Nordheim (FN) operation and use a Ω-gate structure to have little deterioration. These p-channel twin fin field-effect transistor (FinFET) EEPROM devices with a Ω-gate structure have excellent retention and endurance.

## Methods

**Figure 1:**
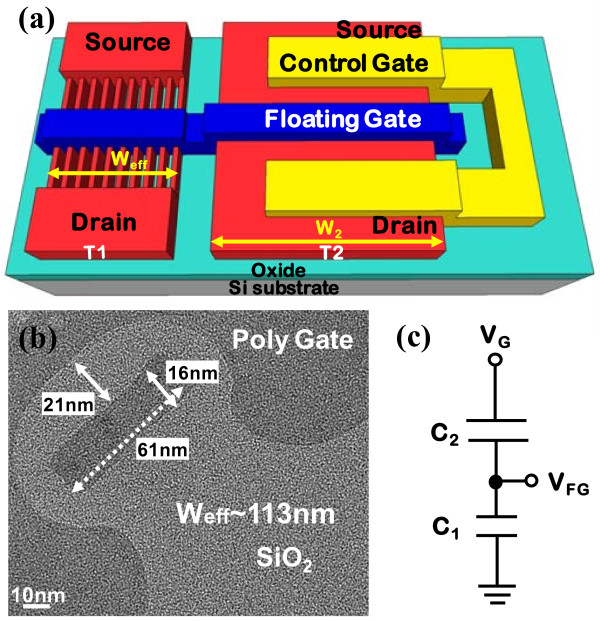
**Schematic, TEM image, and equivalent circuit of twin poly-Si TFT EEPROM. (a)** Schematic of the twin poly-Si TFT EEPROM cell with ten NWs. **(b)** The TEM image of Ω-gate NW twin poly-Si TFT EEPROM. The effective channel width is 113 nm × 10 [(61 nm + 16 nm × 2 + 10 nm × 2) × 10)]. **(c)** The equivalent circuit of twin poly-Si TFT EEPROM.

First, a p-type undoped channel twin poly-Si TFT EEPROM with ten NWs was fabricated. Figure 1a presents the structure of the NW twin poly-Si TFT EEPROM. The gate electrodes of two TFTs are connected to form the floating gate, while the source and drain of the larger TFT (T2) are connected to form the control gate. Figure 1b presents the transmission electron microscopy (TEM) image of the NW EEPROM perpendicular to the gate direction; the NWs are surrounded by the gate electrode as a Ω-gate structure with an effective width of 113 nm.

These devices were fabricated by initially growing a 400-nm-thick thermal oxide layer on 6-in. silicon wafers as substrates. A thin 50-nm-thick undoped amorphous Si (a-Si) layer was deposited by low-pressure chemical vapor deposition (LPCVD) at 550°C. The deposited a-Si layer was then solid-phase-crystallized at 600°C for 24 h in nitrogen ambient. The device’s active NWs were patterned by electron beam (e-beam) direct writing and transferred by reactive-ion etching (RIE). Then, they were dipped into HF solution for 60 s to form the Ω-shaped structure. For gate dielectric, a 15-nm-thick layer of thermal oxide was grown as tunneling oxide. Then, a 150-nm-thick poly-Si layer was deposited and transferred to a floating gate by electron beam direct writing and RIE. Then, the T1 and T2 self-aligned P^+^ source/drain and gate regions were formed by the implantation of BF_2_ ions at a dose of 5 × 10^15^ cm^−2^. The dopant was activated by ultrarapid thermal annealing at 1,000°C for 1 s in nitrogen ambient. Then, a 200-nm-thick TEOS oxide layer was deposited as the passivation layer by LPCVD. Next, the contact holes were defined and 300-nm-thick AlSiCu metallization was performed. Finally, the devices were then sintered at 400°C in nitrogen ambient for 30 min.

In programming, the electrons tunnel into T1 through the tunneling oxide. The tunneling oxide of NW-based EEPROM is surrounded by the gate electrode (Figure 1b). Figure 1c shows the equivalent circuit of this twin TFT NVM:

(1) $$\begin{array}{l} V_{\mathrm{FG}}=\left[C_{2}/\left(C_{{}_{1}}+C_{2}\right)\right]\times V_{G}\\ \;=\left[W_{2}/\left(W_{1}+W_{2}\right)\right]\times V_{G}=\alpha_{G}\times V_{G}. \end{array}$$

To maximize the voltage drop in the tunnel oxide of T1, the gate capacitance of T2 (C_2_) must exceed the gate capacitance of T1 (C_1_). Hence, the NVM device with a high *α*_G_ exhibits a high P/E speed and can be operated at a low voltage. In this work, the devices were designed to have a coupling ratio of 0.85, which is extremely high for memory applications.

## Results and discussion

**Figure 2:**
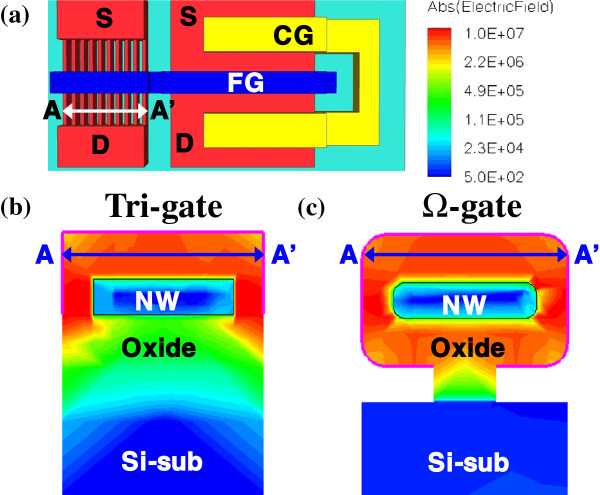
**Electric field of NWs.** By TCAD simulation, cut from the AA’ line in the **(a)** schematic, the electric field around the NWs of **(b)** tri-gate and **(c)** Ω-gate structures is shown.

The TEM image in Figure 1b shows the rounded corners of the twin TFT device structure. First, the NW tri-gated structure, formed by e-beam lithography, was dipped into DHF solution, forming rounded corners. Then, thermal oxidation was performed to form the tunneling oxide; the junction of the channel and the tunneling oxide exhibits some rounding, protecting the tunneling oxide against excessive damage when it is written and erased. The P/E speed and reliability are balanced by Ω-gate formation. By *technology computer-aided design* (TCAD) simulation, Figure 2 shows the electric field of NWs using tri-gate and Ω-gate structures. The result indicates that the Ω-gate structure has more programming sites around the NWs than the tri-gate structure which are only at the upper corners and that the Ω-gate structure also has smoother electric field.

**Figure 3:**
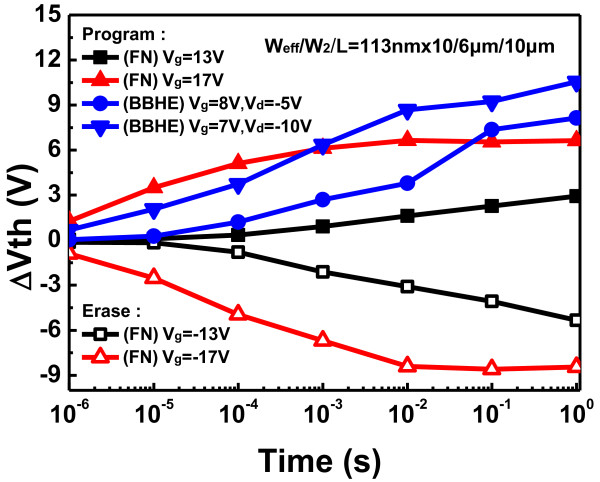
**Programming and erasing characteristics of the EEPROM cell with devices.** The P/E speed of BBHE operation is compared with that of FN operation.

Figure 3 compares the P/E speed of the BBHE operation with that of the FN operation. The device was programmed by FN injection at *V*_gs_ = 17 V and by BBHE injection at *V*_gs_ = 7 V with *V*_ds_ = −10 V. The BBHE operation exhibits higher programming speed than the FN operation.

**Figure 4:**
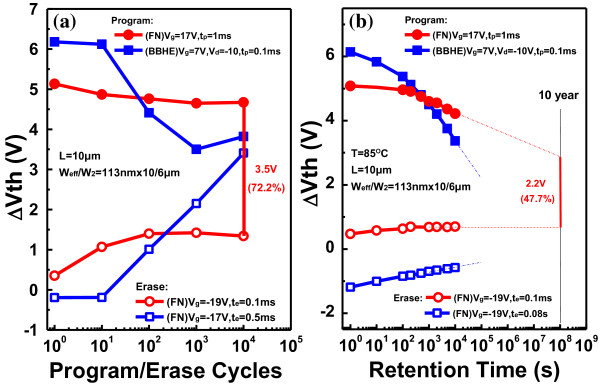
**Endurance and retention characteristics. (a)** Endurance characteristics of the twin poly-Si TFT EEPROM by FN and BBHE. **(b)** Retention characteristics of the twin poly-Si TFT EEPROM at 85°C by FN and BBHE.

Figure 4a shows the twin poly-Si TFT-based (*W*_eff_/*W*_2_/*L* = 113 nm × 10/6 μm/10 μm) EEPROM P/E cycling endurance characteristics by FN and BBHE, respectively, using the same input voltage. As the number of P/E cycles increased, the magnitude of the memory window disappeared. The floating-gate memory device maintained a wide threshold voltage window of 3.5 V (72.2%) after 10^4^ P/E cycles for FN operation. For BBHE operation, the memory window was almost closed after 10^4^ P/E cycles. Figure 4b shows high-temperature (85°C) retention characteristics of NW-based (*W*_eff_/*W*_2_/*L* = 113 nm × 10/6 μm/10 μm) EEPROMs. This figure reveals that after 10 years, the memory window was still 2.2 V when using FN operation. For BBHE operation, the device exhibited almost no data retention capacity. The Ω-gate structure has a higher P/E efficiency than the tri-gate structure because the four corners of the channel are all surrounded by the gate structure [13,14]. The Ω-gate structure contributes to the equal sharing of the electric field and reduces the probability of leakage in the floating-gate devices in the form of stress-induced leakage current, improving the reliability of the device. Also, the extra corners improve the P/E speed.

**Figure 5:**
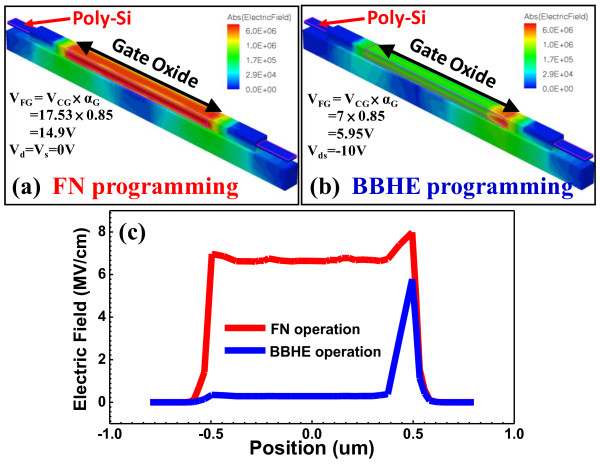
**TCAD simulation. (a)** FN programming. *V*_FG_ = *V*_CG_ × *α*_G_ = 14.9 V. **(b)** BBHE programming. *V*_FG_ = V_CG_ × *α*_G_ = 5.95 V. Both use the same voltage drop. **(c)** Electric field comparison of FN and BBHE programming.

Figure 5 displays a TCAD simulation of FN and BBHE operations. The result indicates that the FN operation produces a high average electric field in the tunneling oxide from the source to the drain, programmed by the tunneling effect. FN operation indicates the average wearing of electric field on the tunneling oxide. BBHE operation produces a sudden electric field peak at the source side, programmed using hot electrons with high energy, causing considerable local damage to the tunneling oxide. This result of consistent P/E that is caused by FN operation reveals better endurance and retention than the BBHE operation for floating-gate devices.

## Conclusions

This work developed a novel Ω-gate NW-based twin poly-Si TFT EEPROM. Experimental results demonstrated that the Ω-gate NW-based structure had a large memory window and high P/E efficiency because of its multi-gate structure and even oxide electrical field at the NW corners. After 10^4^ P/E cycles, Δ*V*_th_ = 3.5 V (72.2%). The proposed twin-TFT EEPROM with a fully overlapped control gate exhibited good data endurance and maintained a wide threshold voltage window even after 10^4^ P/E cycles. This Ω-gate NW-based twin poly-Si TFT EEPROM can be easily incorporated into an AMLCD array press and SOI CMOS technology without any additional processing.

## Competing interests

The authors declare that they have no competing interests.

## Authors’ contributions

M-SY and M-FH carried out the device mask layout, modulated the coupling ratio of the device, handled the experiment, and drafted the manuscript. K-CL measured the characteristics of the device and made the simulation plot. Y-RJ and L-CC gave some physical explanation to this work. Y-CW conceived the idea of low-temperature deposition of twin FinFET and their exploitation into devices. He also supervised the work and reviewed the manuscript. C-YC participated in the design and coordination of the study. All authors read and approved the final manuscript.
